# Inclusion Body Myositis With Dysphagia Diagnosed Using Pharyngoesophageal High-Resolution Manometry

**DOI:** 10.14309/crj.0000000000000850

**Published:** 2022-08-31

**Authors:** Toshimi Chiba

**Affiliations:** 1 Division of Internal Medicine, Department of Oral Medicine, Iwate Medical University, Morioka, Iwate, Japan

## CASE REPORT

A 68-year-old woman developed progressive dysphagia with a 5 kg weight loss over 2 years. Upper gastrointestinal endoscopy showed no mucosal lesions in the laryngopharynx and esophagus and no increased resistance at the esophagogastric junction. No nasal speech or hoarseness was noted. No extremity muscle weakness was apparent, and all manual muscle strength tests were normal. Computed tomography findings showed no atrophic changes in the quadriceps femoris muscles. The serum creatinine kinase level was slightly elevated, and a needle electromyogram of the right vastus intermedius muscle revealed myopathic potentials. A muscle biopsy was obtained from the left rectus femoris; the pathologic evaluation was consistent with inclusion body myositis (IBM).

**Figure 1.**
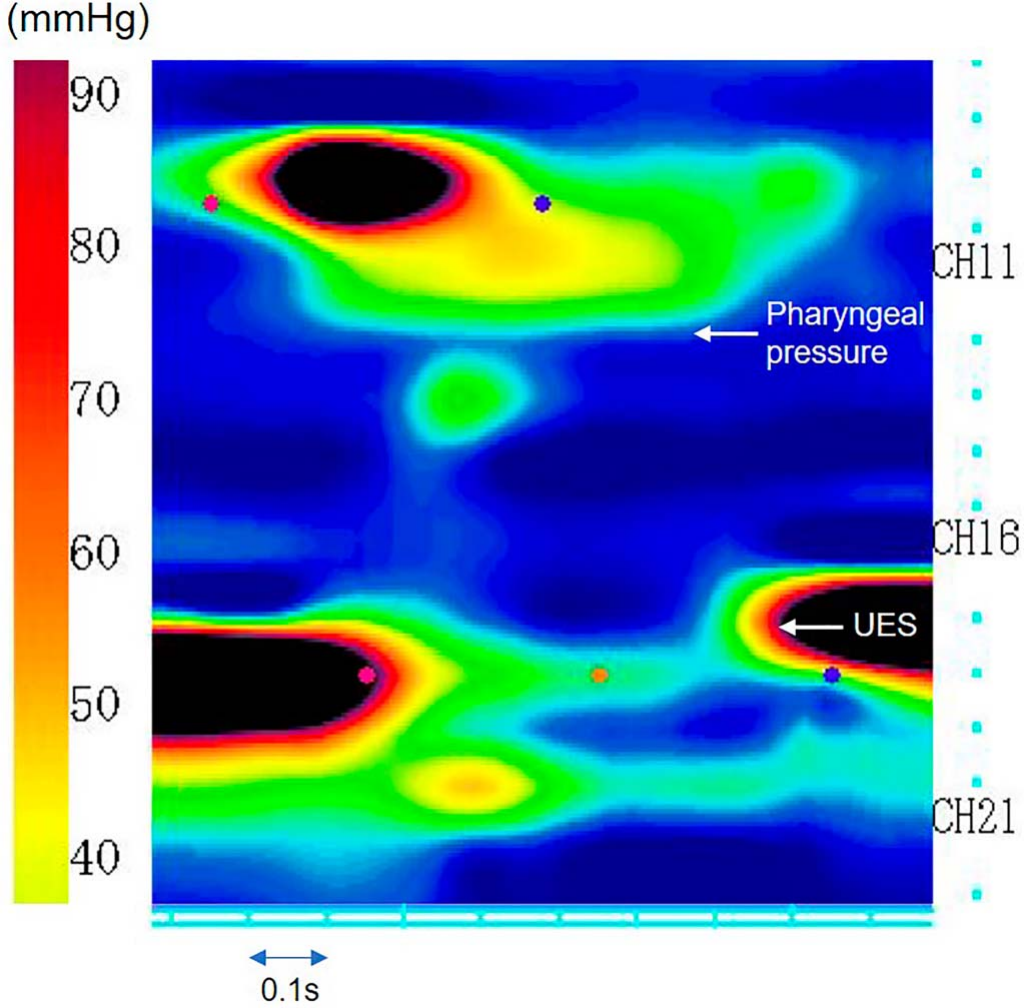
Pharyngoesophageal high-resolution manometry. Pharyngoesophageal high-resolution manometry showed weak pharyngeal pressure, slow pharyngeal velocity to the UES, and positive UES relaxation pressure. UES, upper esophageal sphincter.

**Figure 2.**
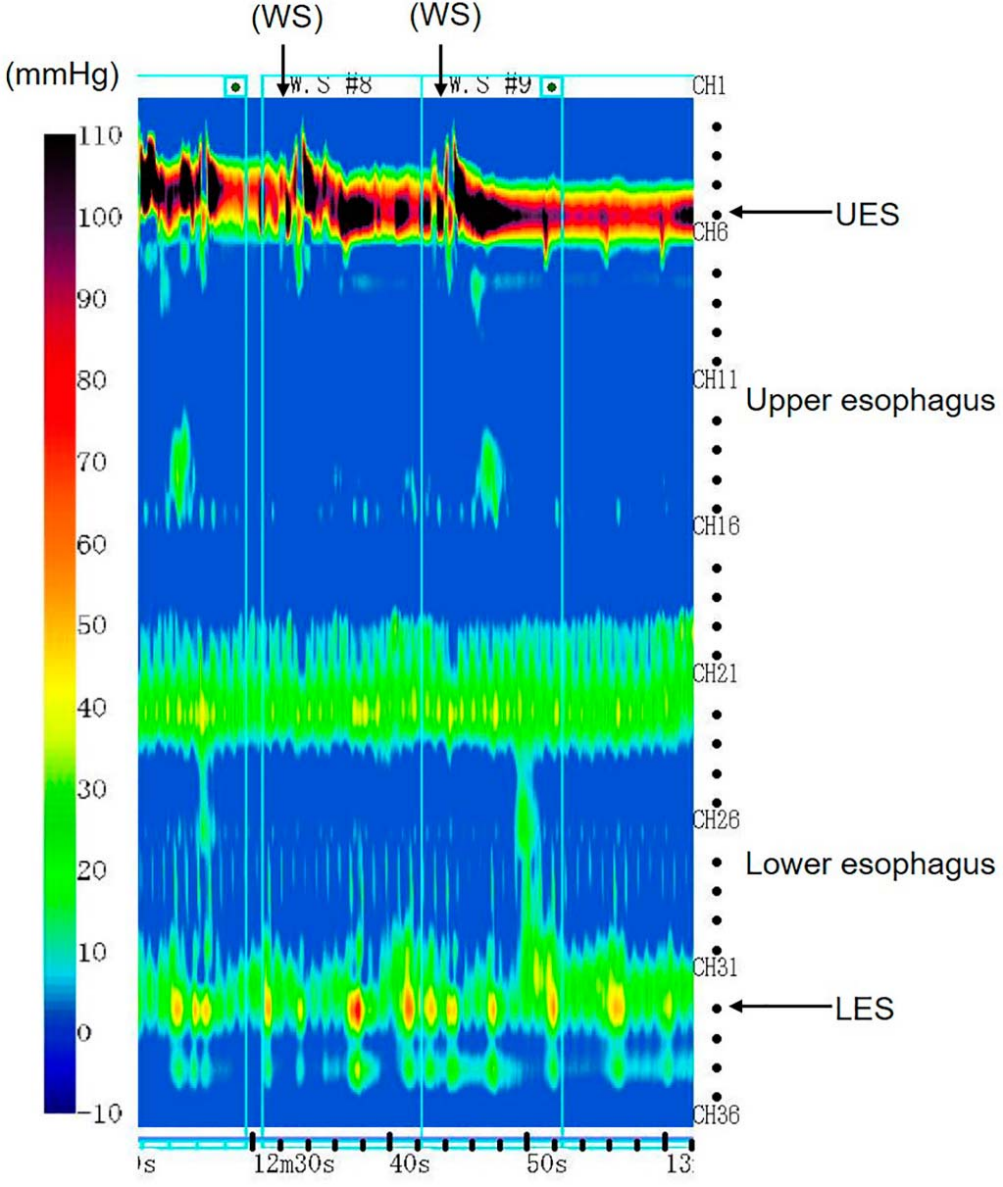
Esophageal high-resolution manometry. Esophageal high-resolution manometry showed high integrated relaxation pressure, low distal contractile integral, and normal lower esophageal sphincter pressure; an absence of peristalsis in the esophagus was demonstrated. LES, lower esophageal sphincter; UES, upper esophageal sphincter; WS, wet swallow.

Pharyngoesophageal high-resolution manometry (HRM) was performed for investigation of progressive dysphagia. HRM showed weak pharyngeal pressure (25.2 ± 9.8 mm Hg; normal mean = 71.7 mm Hg), slow pharyngeal velocity to the upper esophageal sphincter (1.2 ± 1.7 cm/s; normal mean = 3.5 cm/s), and positive upper esophageal sphincter relaxation pressure (4.7 ± 7.9 mm Hg; normal mean = –6.11 mm Hg), all of which indicated pharyngeal dysmotility (Figure 1).^1,2^ Esophageal HRM showed high integrated relaxation pressure (16.8 ± 6.6 mm Hg; normal mean = 6 mm Hg), low distal contractile integral (103.7 ± 52.4 mm Hg-cm-s; normal mean = 1868.25 mm Hg-cm-s), normal lower esophageal sphincter pressure (26.8 ± 4.4 mm Hg; normal mean = 18.95 mm Hg), and absence of peristalsis in the esophagus (Figure 2).^2^

Sporadic IBM (sIBM) is a chronic progressive muscular disorder that develops in people aged 50 years and older. Asymmetric muscle weakness and amyotrophy of the quadriceps femoris muscle and a finger or wrist flexor often accompany sIBM. Histopathologic analysis of skeletal muscle biopsies from patients with sIBM reveals rimmed vacuoles and an inflammatory cell infiltration.^3^ Dysphagia has been reported in 40%–80% of patients with sIBM.^4^ The causes of dysphagia in patients with sIBM are related to inadequate pharyngeal contraction, poor relaxation of the cricopharyngeal muscle, and reduced hyolaryngeal elevation. The pharyngoesophageal muscle tone is lost; therefore, patients develop nasal speech, hoarseness, nasal regurgitation, and aspiration pneumonia.^5^ In our case, dysphagia without nasal speech and hoarseness was observed; however, pharyngoesophageal HRM revealed pharyngeal dysmotility, which indicates a reduction in propulsion of a bolus through the sphincter muscles. Subsequently, the absence of esophageal peristalsis was also demonstrated, which can be an induced problem with a lack of initiation of the propagation.

## DISCLOSURES

Author contributions: T. Chiba wrote and edited the manuscript and is the author guarantor.

Financial disclosure: None to report.

Previous presentation: This case was presented at the Japanese Society of Neurogastroenterology meeting; October 7-8, 2021; Sendai, Japan.

Informed consent was obtained for this case report.
